# Current State of the Fight Against Antimicrobial Resistance: What Are the Different Strategies for Tomorrow?

**DOI:** 10.3390/antibiotics15060564

**Published:** 2026-06-01

**Authors:** Hicham Wahnou, Riad El Kebbaj, Béatrice Demoré, Youness Limami, Raphaël Emmanuel Duval

**Affiliations:** 1 Sciences and Engineering of Biomedicals, Biophysics and Health Laboratory, Higher Institute of Health Sciences, Hassan First University, Settat 26000, Morocco; elkebbajriad@gmail.com (R.E.K.); youness.limami@uhp.ac.ma (Y.L.); 2 Mohammed VI Center for Research & Innovation (CM6RI), Rabat 10112, Morocco; 3 Pharmacy Department, University Hospital, Allée du Morvan, 54511 Vandœuvre-lès-Nancy, France; b.demore@chru-nancy.fr; 4 INSPIIRE, Inserm, Université de Lorraine, 54000 Nancy, France; 5 Université de Lorraine, 54000 Nancy, France

**Keywords:** antimicrobial resistance, antibiotic discovery, drug-resistant infections, global health policy

## Abstract

Antimicrobial resistance (AMR) is a leading global cause of death, with recent World Health Organization (WHO) data revealing that one in six laboratory-confirmed bacterial infections shows resistance to at least one antibiotic treatment. This review comprehensively analyzes the AMR landscape in 2026, detailing its evolution, mechanisms, and the innovative strategies being deployed to combat it. Driven by Darwinian selection and accelerated by factors like antibiotic overuse during the Coronavirus Disease 2019 (COVID-19) pandemic (predominantly in hospitalized patients with suspected bacterial co-infection), AMR is propelled by a diverse molecular arsenal in bacteria. Key mechanisms include enzymatic drug inactivation (e.g., the diversifying β-lactamase superfamily), target site modification (e.g., *mcr* genes conferring colistin resistance), efflux pumps, and biofilm formation. The rapid global spread of these traits is facilitated by a dynamic “mobilome”, a network of plasmids and transposons that shuttle resistance genes between species. This crisis has sparked a major scientific mobilization. Advances include the discovery of novel antibiotic scaffolds like lariocidin and the regulatory approval of critical new antibiotic/inhibitor combinations such as sulbactam/durlobactam and aztreonam/avibactam, which target highly resistant Gram-negative bacteria. Moreover, the first-in-class antibiotic gepotidacin offers a new option for urinary tract infections. Beyond traditional drugs, the pipeline is diversifying to include phage therapy, antivirulence strategies, and artificial intelligence-guided drug discovery. This diversification is critical as it helps preserve the effectiveness of existing Medically Important Antimicrobials (MIAs), those deemed essential for human medicine, by providing alternative or adjunctive treatment options. However, scientific innovation alone is insufficient. This review argues that lasting success requires parallel progress in global policy and infrastructure. Strategic priorities beyond 2026 must include finalizing and funding updated global action plans, strengthening real-time surveillance and diagnostic capacity, especially in low-resource settings, and implementing new economic models to de-risk antibiotic development. Embedding effective antimicrobial stewardship within universal health coverage and pandemic preparedness plans is crucial. Ultimately, defeating AMR demands an unprecedented, coordinated global effort that outpaces the relentless adaptability of bacterial pathogens.

## 1. Introduction

**Figure 1:**
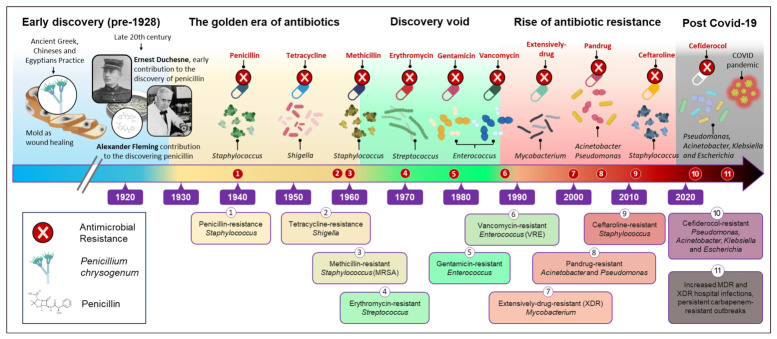
Presentation of a dual-axis timeline illustrating the parallel evolution of antibiotic discovery (top axis, from ancient practices to modern drugs) and the corresponding emergence of antimicrobial resistance (bottom axis, from early resistance to modern pandrug resistance). It visually maps the historical relationship between key therapeutic milestones, such as the discovery of penicillin, tetracycline, and vancomycin; and the subsequent development of resistant bacterial pathogens, including Methicillin-Resistant *Staphylococcus aureus* (MRSA), Vancomycin-Resistant *Enterococcus* (VRE), and Extensively Drug-Resistant/Pandrug-Resistant (XDR/PDR) strains of *Pseudomonas*, *Acinetobacter*, and *Mycobacterium tuberculosis*. The graphic highlights the ongoing challenge of antimicrobial resistance, extending into the post-COVID-19 era with newer agents and persistent multidrug-resistant outbreaks.

Since the introduction of penicillin in 1941, antibiotics have revolutionized clinical medicine, dramatically reducing morbidity and mortality from bacterial infections. Yet, only a few years after its deployment, *Staphylococcus aureus* isolates exhibiting penicillinase activity (i.e., penicillin resistance) emerged, an early warning of bacterial adaptability [1]. The following decades witnessed successive waves of resistance, each following the introduction of a new antibiotic class (Figure 1). The phenomenon is driven by Darwinian selection: exposure to antimicrobial compounds imposes selective pressure, allowing resistant genotypes to proliferate within bacterial populations [2].

Antimicrobial resistance (AMR) is now recognized as a leading cause of death worldwide. The 2025 WHO report revealed that resistant infections cause an estimated 1.3 million direct deaths each year, and indirectly contribute to over 5 million more. This figure is comparable to the number of deaths caused by cancer [3]. Particularly alarming is the rise of resistance among the “ESKAPE” pathogens, *Enterococcus faecium*, *Staphylococcus aureus*, *Klebsiella pneumoniae*, *Acinetobacter baumannii*, *Pseudomonas aeruginosa*, and *Enterobacter* spp., which collectively account for the majority of healthcare-associated infections (HAIs) [4,5]. Multidrug-resistant (MDR) and extensively drug-resistant (XDR) strains now complicate treatment of community and hospital infections alike, escalating healthcare costs and hospital stays [3].

The mechanistic basis of resistance is multifactorial. Bacteria may enzymatically inactivate antibiotics (e.g., β-lactamases), modify drug targets (e.g., methylation of 23S rRNA), reduce intracellular drug accumulation via efflux pumps, or develop biofilm-based protection [6]. Gene exchange through MGEs (mobile genetic elements, e.g., plasmids, transposons, and integrons) accelerates the dissemination of resistance determinants, forming vast “resistomes” within microbial communities [7]. Genomic surveillance over the past five years has revealed that such genetic plasticity is not confined to hospitals: resistant determinants are now widespread in commensal and environmental microbiota [8].

The COVID-19 pandemic left a lingering legacy of antibiotic overuse. Between 2020 and 2023, more than 70% of hospitalized COVID-19 patients received empirical antibiotics, despite the viral etiology of the primary infection, accelerating selective pressure in both community and healthcare settings [9]. By 2025, surveillance data confirmed rising resistance to fluoroquinolones, macrolides, and third-generation cephalosporins in multiple regions [10,11]. The persistence of these prescribing behaviours underscores systemic weaknesses in antimicrobial stewardship programs.

The past two years have seen unprecedented scientific and institutional mobilization. On the research front, new antibiotic scaffolds such as lariocidin, a lasso peptide characterized in 2025 with a novel mechanism of action against Gram-negative bacteria (GNB), signal renewed interest in non-traditional molecules [12]. Artificial intelligence and genome mining have accelerated the identification of cryptic biosynthetic gene clusters, reviving natural-product discovery [13]. Meanwhile, synthetic biology and Clustered Regularly Interspaced Short Palindromic Repeats (CRISPR)-based strategies are being developed to selectively disable resistance genes or re-sensitize pathogens to existing drugs [14].

On the policy front, WHO’s Global Action Plan on AMR entered a new revision cycle (2025–2027), reinforcing multisectoral coordination under the One Health framework. The 2025 edition of the Tracking Antimicrobial Resistance Country Self-Assessment Survey (TrACSS) achieved record participation from 163 countries, signalling improved surveillance capacity [3]. Additionally, the updated AWaRe classification emphasizes shifting national antibiotic consumption toward “Access” agents, discouraging overuse of “Watch” and “Reserve” groups [15].

This review aims to synthesize these scientific, clinical, and policy developments to assess whether the global response to AMR is truly accelerating. It will evaluate if recent progress in novel antibiotic discovery, alternative therapies, and strengthened global governance represents a decisive turning point, or if the relentless evolution of bacterial resistance continues to outpace our collective efforts.

## 2. Evolution of Antibiotic Resistance Mechanisms

### 2.1. Overview of Adaptive and Genetic Evolution

Antibiotic resistance emerges through both vertical evolution, where spontaneous mutations provide a selective advantage under drug pressure, and horizontal gene transfer (HGT), which disseminates resistance determinants across species boundaries [16]. While mutation-driven adaptation is dominant in chronic infections where a single bacterial strain persists and evolves within a host, such as in *P. aeruginosa* populations in the lungs of people with cystic fibrosis, or in *M. tuberculosis* during tuberculosis treatment, where resistance to key drugs like rifampicin often arises from point mutations in the target genes (e.g., *rpoB*); HGT through MGEs has been the principal driver of the rapid global proliferation of resistance genes across diverse bacterial populations and settings [7]. Classical examples of mutation-mediated resistance also include fluoroquinolone resistance resulting from mutations in genes encoding DNA topoisomerases (e.g., *gyrA*, *parC*), and oxazolidinone resistance linked to 23S rRNA gene mutations [17,18]. Since 2020, genomic and metagenomic analyses have shown that most clinically significant resistance determinants originate in environmental and commensal reservoirs, including soil, aquatic microbiomes, and the human gut [19].

**Figure 2:**
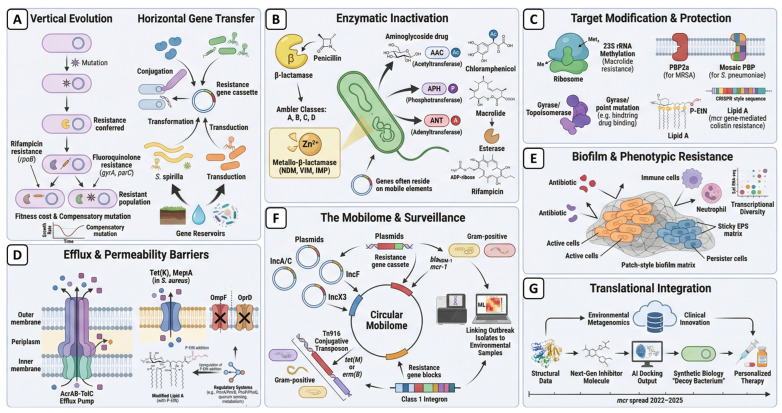
The multifaceted mechanisms of antimicrobial resistance, organized into thematic panels. (**A**) Vertical evolution through mutation and selection, and Horizontal gene transfer via mobile genetic elements. (**B**) Enzymatic inactivation of drugs. (**C**) Target modification and protection. (**D**) Efflux pumps and permeability barriers. (**E**) The mobilome and outbreak surveillance. (**F**) Phenotypic resistance in biofilms. (**G**) Translational integration of environmental data into clinical innovation.

The evolutionary dynamics of resistance are further shaped by fitness costs and compensatory mutations. Although resistance mutations often reduce bacterial fitness, compensatory changes can restore growth rates while retaining resistance, stabilizing these traits even in the absence of antibiotics [20]. Experimental evolution and long-term sequencing of resistant *Escherichia coli* and *K. pneumoniae* populations from 2017–2021 demonstrated that compensatory epistasis has become a crucial factor sustaining multidrug resistance [21] (Figure 2A).

### 2.2. Enzymatic Degradation: The Expanding β-Lactamase Family and Beyond

Among enzymatic mechanisms, β-lactamases remain the most clinically consequential. These enzymes hydrolyse the β-lactam ring, neutralizing penicillins, cephalosporins, carbapenems, and monobactams (Figure 2B). They are classified into Ambler classes A–D based on molecular structure and catalytic activity [22]. The last decade has witnessed a diversification of Metallo-β-Lactamases (MβLs) such as New Delhi Metallo-β-lactamase (NDM), Verona Integron-encoded Metallo-β-lactamase (VIM), and Imipenemase Metallo-β-lactamase (IMP) variants, which use zinc to catalyse hydrolysis and are unaffected by most commercial inhibitors [23]. The emergence of NDM-33, first detected in 2024 in India and now widespread across South Asia and the Middle East, represents a particularly troubling adaptation [3,10]. Mechanistically, NDM-33 differs from the prevalent NDM-5 variant by a single amino acid substitution (A72T) [24]. Biochemical characterization reveals that NDM-33 exhibits enhanced hydrolytic activity against meropenem, with a k_cat_/K_m_ of 3.24 μM^−1^ s^−1^ compared to 1.96 μM^−1^ s^−1^ for NDM-5, while showing reduced activity against ceftazidime and cefepime [24]. The blaNDM-33 gene is carried on an IncX3-type plasmid within a Tn125-related genetic context, facilitating horizontal transfer [24]. To date, high-resolution crystallographic data for NDM-33 have not been published, limiting detailed structural comparison with NDM-1 or NDM-5.

Among Gram-positive bacteria (GPB), the acquisition of staphylococcal penicillinases (*blaZ*) remains a cornerstone of penicillin resistance in *S. aureus*, while altered Penicillin-Binding Proteins (PBPs), particularly PBP2a in methicillin-resistant *S. aureus* (MRSA) and mosaic PBPs in ceftriaxone-resistant *Streptococcus pneumoniae*, exemplify target-based resistance mechanisms that are often plasmid-encoded or arise through homologous recombination [25].

Class D enzymes, notably OXA-type carbapenemases, have also expanded in *A. baumannii* and Enterobacterales, often carried on mobile plasmids with hybrid resistance modules combining β-lactamase and efflux pump genes [26]. Even in the face of new inhibitors such as avibactam or relebactam, bacteria are evolving substitutions that restore enzyme function, an example of evolutionary reversibility under drug pressure [23].

Beyond carbapenemase production (e.g., OXA-23, OXA-24/40), Carbapenem-resistant *Acinetobacter baumannii* (CRAB) resistance is driven by (i) upregulation of the AdeABC efflux pump system, which extrudes tigecycline and fluoroquinolones; (ii) loss or downregulation of CarO and OprD porins, reducing carbapenem influx; and (iii) lipid A modifications via LpxL/LpxM mutations, conferring colistin resistance. These mechanisms frequently co-occur, creating a synergistic multidrug-resistant phenotype [27,28]. (See also Section 4.2 for therapeutic implications.)

While β-lactamases represent the most diverse and clinically dominant family of resistance enzymes, they are by no means the only enzymatic route for antibiotic inactivation. Bacteria have evolved a vast arsenal of transferases and modifying enzymes that specifically target other major antibiotic classes, further limiting therapeutic options.

Aminoglycoside-modifying enzymes (AMEs) are a prime example. These enzymes inactivate aminoglycosides (e.g., gentamicin, amikacin) through three principal mechanisms: acetylation (acetyltransferases, AACs), phosphorylation (phosphotransferases, APHs), and adenylation (nucleotidyltransferases, ANTs) [29]. The genes encoding these enzymes are frequently located on MGEs, facilitating their rapid spread among GNB and GPB [29,30]. The increasing prevalence of AMEs contributes significantly to the loss of efficacy of this important antibiotic class, particularly in hospital-acquired infections.

Another major group comprises chloramphenicol acetyltransferases (CATs), which inactivate chloramphenicol by acetylation, and esterases that target macrolides (e.g., Ere enzymes conferring resistance to erythromycin) [31]. Furthermore, rifampicin-modifying enzymes such as rifampin ADP-ribosyltransferases (e.g., Arr) can chemically modify and inactivate this key anti-tuberculosis drug, posing a threat to treatment of mycobacterial infections [32].

The diversity of these enzymatic mechanisms, often operating in concert within the same bacterial cell, underscores the immense adaptive capacity of bacteria and highlights the need for combination therapies and inhibitors with broader specificity.

### 2.3. Target Modification and Protection

Target modification confers resistance by altering or shielding the molecular binding site of antibiotics. Common examples include methylation of 23S rRNA (macrolides, lincosamides), mutation of *gyrA*/*parC* genes (fluoroquinolones), and alteration of penicillin-binding proteins (β-lactams) [33]. Since 2023, genomic surveillance has revealed new mosaic PBPs in *S. pneumoniae* that confer reduced susceptibility to ceftriaxone without incurring major fitness costs [34].

A particularly concerning trend is the global spread of mobilized colistin resistance (*mcr*) genes, which modify lipid A via phosphoethanolamine transferases, conferring resistance to colistin, an antibiotic of last resort [35]. Between 2022 and 2025, ten distinct *mcr* variants (*mcr-1* to *mcr-10*) were reported, with *mcr-9* now endemic in several European hospital networks [3]. This exemplifies the continuing adaptability of bacterial cell envelope targets under intense pharmacological pressure (Figure 2C).

### 2.4. Efflux Pumps and Permeability Barriers

Efflux-mediated resistance, particularly in GNB, represents a formidable barrier to antibiotic efficacy. These multidrug efflux systems, such as AcrAB-TolC (Enterobacterales) and MexAB-OprM (*P. aeruginosa*), actively export structurally diverse compounds including β-lactams, macrolides, tetracyclines, and fluoroquinolones [36]. In GPB, efflux pumps also play a significant role. The Tet(K) and Tet(L) proteins, for example, are tetracycline-specific exporters commonly found in staphylococci and enterococci [37]. Similarly, the MepA pump in *S. aureus* contributes to reduced susceptibility to tigecycline and fluoroquinolones [37]. Recent proteomic studies (2024–2025) have elucidated regulatory networks linking efflux expression with quorum sensing and metabolic state, indicating that efflux is not merely a defensive trait but part of a broader adaptive physiology [38,39,40] (Figure 2D).

Parallel to efflux, porin loss and membrane remodelling reduce antibiotic entry. In fact, the deletion or mutation of porins, such as OmpF and OprD drastically decreases antibiotic uptake. In *A. baumannii* and *K. pneumoniae*, OprD loss specifically reduces carbapenem uptake, while modification to lipid A reduces polymyxin binding [41,42,43]. Combined with efflux, these mechanisms create formidable “permeability shields” (Figure 2D).

### 2.5. Genomic Surveillance and the Rise of the “Mobilome”

In recent years, metagenomic and plasmidomic surveillance reshaped our understanding of AMR evolution. Whole-genome sequencing (WGS) data integrated into the Global Antimicrobial Resistance Surveillance System (GLASS) and regional networks (e.g., EARS-Net, Africa CDC AMRS) revealed that most resistance genes circulate within complex mobile gene cassettes shared among environmental and clinical bacteria [3]. The term “mobilome” now encapsulates this dynamic ensemble of MGEs mediating gene flow across species [44] (Figure 2E).

For example, the global dissemination of NDM-1, first identified in 2008, was driven almost entirely by promiscuous Inc-type plasmids (e.g., IncA/C, IncF, IncX3) that shuttled the *blaNDM-1* gene across Enterobacterales species and into diverse geographic regions [45]. Similarly, the *mcr-1* gene conferring colistin resistance emerged on an IncI2 plasmid in China before rapidly spreading to five continents via multiple plasmid backbones [46]. In GPB, the Tn916 family of conjugative transposons has played a central role in disseminating tetracycline (tet(M)) and macrolide (erm(B)) resistance genes among streptococci, staphylococci, and enterococci [47]. Integrons, particularly class 1 integrons, act as gene capture and expression systems, assembling resistance gene cassettes (e.g., *aadB*, *dfrA*) into arrays that can be mobilized by insertion sequences and transposons [48].

This ever-expanding mobilome blurs the boundaries between environmental, animal, and clinical resistomes, creating a shared genetic reservoir that fuels the rapid adaptation of pathogens to antimicrobial pressure.

Novel computational pipelines, including machine learning classifiers trained on metagenomic datasets, have enabled prediction of resistance determinants from uncharacterized microbiomes [49]. Such tools have already been deployed in hospitals for real-time outbreak tracing, linking resistance plasmids between wastewater and patient isolates [50]. This represents a major advance in containment strategies, providing molecular epidemiology capable of anticipating rather than reacting to AMR dissemination.

### 2.6. Biofilm-Mediated and Phenotypic Resistance

The extracellular polymeric substance (EPS) matrix, composed of polysaccharides, eDNA, and proteins, acts as a diffusion barrier and sequesters antibiotics [51]. Metabolic heterogeneity within biofilms, driven by oxygen and nutrient gradients, generates persister subpopulations that tolerate high antibiotic concentrations [51]. Biofilm formation provides collective protection, creating physical barriers and metabolic heterogeneity that reduce antibiotic penetration [52]. Within biofilms, subpopulations known as persister cells enter dormant states refractory to most antibiotics [53]. This phenomenon is particularly problematic in GNB such as *P. aeruginosa*, a classic model organism for biofilm research, where alginate-overproducing mucoid strains in the lungs of cystic fibrosis patients create chronic, virtually impenetrable biofilm communities that resist both antibiotics and host immune clearance [54]. Similarly, biofilms formed by *A. baumannii* on medical devices contribute to its persistence in hospital environments [54]. Furthermore, recent single-cell RNA-seq studies revealed extensive transcriptional heterogeneity within *S. aureus* biofilms, uncovering distinct metabolic, stress-response, and virulence-related subpopulations and mapping regulatory trajectories from planktonic to biofilm states, thereby highlighting persistence-associated adaptations under immune and environmental pressures [55]. Furthermore, *P. aeruginosa* employs quorum sensing to coordinate biofilm maturation and virulence factor expression [56]. These insights underscore that eradicating infection requires not only targeting active resistance genes but also disrupting bacterial dormancy networks. Clinical data further corroborate these findings. The previous study reported that clinical *E. coli* isolates from urinary tract infections were capable of biofilm formation, with over 44% exhibiting strong biofilm production. Importantly, strong biofilm producers showed significantly higher resistance rates to various antibiotics, empirically establishing a quantitative relationship between biofilm strength and antibiotic resistance profiles at the clinical level [57] (Figure 2F).

### 2.7. Integrating Molecular Mechanisms into Therapeutic Design

Understanding the molecular basis of resistance has direct translational value. Structure-guided design of next-generation inhibitors (e.g., zidebactam, nacubactam) specifically targets resistant β-lactamases, while AI-guided molecular docking predicts efflux avoidance profiles [58,59,60]. Synthetic biology platforms have begun engineering bacterial “decoy” systems that competitively sequester resistance genes, though these remain experimental [61]. The intersection of genomics, structural biology, and computational design thus marks a new era of precision antimicrobial therapy, moving beyond empirical regimens toward personalized infection management (Figure 2G).

Recent advances in bioinformatics and large-scale genomic data mining have significantly enhanced our understanding of the evolutionary origins and dissemination pathways of antimicrobial resistance genes (ARGs) [62]. Metagenomic analyses have revealed that many clinically relevant resistance determinants predate the antibiotic era and originated in environmental bacteria before being mobilized into pathogenic species [63]. For instance, extended-spectrum β-lactamase genes such as *blaCTX-M* have been traced back to environmental *Kluyvera* sp., highlighting the importance of ecological reservoirs in shaping modern resistance patterns [64]. Furthermore, computational surveillance of environmental matrices, including wastewater metagenomes, has enabled the early identification of emerging resistance genes such as *mcr-9* prior to widespread clinical dissemination [65]. Integrating data mining approaches with mechanistic microbiology therefore provides a predictive framework for anticipating resistance evolution and rationally guiding the design of next-generation antimicrobials and resistance inhibitors [66].

## 3. The Global State of Antimicrobial Resistance in 2025

### 3.1. Headline Finding: “One in Six” Infections Resistant (GLASS 2025)

The WHO Global Antibiotic Resistance Surveillance Report 2025 (GLASS) synthesized > 23 million bacteriologically confirmed infections reported by 104 countries in 2023 and 110 countries across 2016–2023. The central result is stark: ~1 in 6 laboratory-confirmed bacterial infections in 2023 were resistant to antibiotic treatments, with resistance increasing in >40% of pathogen–antibiotic combinations tracked since 2018 (average rise 5–15%/year) [3]. Independent coverage and summaries from British Medical Journal, Financial Times, and Reuters echoed the same toplines and highlighted geographic heterogeneity (see Section 3.3) [67]. Nevertheless, GLASS data are likely underestimated due to limited laboratory capacity and underreporting in low-resource settings. Conversely, hospital-based surveillance may overestimate resistance in community populations. WHO acknowledges these biases as directional but stable for trend monitoring. Moreover, the “one in six” figure represents resistance prevalence (proportion of tested infections showing resistance), not incidence (rate of new resistant infections per population); the latter requires denominator data on infection rates, which GLASS does not uniformly capture.

### 3.2. Burden: Mortality, Disability-Adjusted Life Years (DALYs), and Health-System Impact

GLASS aggregates resistance prevalence (not mortality) but triangulating with prior Global Burden of Disease-style burden estimates and WHO briefings suggests > 1 million deaths directly attributable to drug-resistant bacterial infections annually and ~5 million associated deaths when considering indirect contributions. These figures remain consistent with earlier global burden modelling and are invoked in WHO’s 2025 communications [67]. While point estimates vary by methodology and input coverage, the directional trend is unambiguous: AMR remains a top-tier cause of death globally and a driver of extended hospital stays and costs, pressures that mount where empiric therapy fails due to resistance to 3rd-generation cephalosporins (3GC), carbapenems, and fluoroquinolones [68]. Resistance to last-line antibiotics (specialized drugs, such as colistin and carbapenems, reserved for treating severe, MDR infections when all other options fail) has reached a critical point in some regions, with rates exceeding 70% for common bacterial pathogens. This alarming finding comes from the WHO GLASS 2025, which identified *E. coli* and *K. pneumoniae* resistant to 3GC as the most pervasive threats in bloodstream and urinary tract infections [3,69]. While global resistance rates were already high, at 44.8% for *E. coli* and 55.2% for *K. pneumoniae*, the African Region faces the direst situation, with over 70% of these infections demonstrating resistance to these essential drugs [3].

### 3.3. Regional Patterns in 2025: Persistent Heterogeneity

GLASS 2025 indicates the highest resistance proportions in South-East Asia and the Eastern Mediterranean (approx. ~1 in 3 infections resistant), while Europe and the Western Pacific are lower (~1 in 10 to ~1 in 11)—though still clinically significant. Africa shows very high resistance to first-line options for certain bloodstream infections, with some settings reporting >70% resistance [3]. Country-level variation is substantial: South Africa (~40% 3GC-R *E. coli*) versus Niger (~70%), reflecting differences in laboratory capacity and antibiotic access. Complementary European surveillance (EARS-Net/Central Asian and European Surveillance of Antimicrobial Resistance (CAESAR)) for 2023 data confirms high and in many cases increasing resistance across key pathogens (e.g., 3GC-resistant (3GC-R) *E. coli/K. pneumoniae*, carbapenem-non-susceptible *Acinetobacter*), sustaining pressure on 2025 front-line practice [3,70]. In Africa, Africa Centres for Disease Control and Prevention (Africa CDC) reports (2024–2025) underscore widespread drug resistance across multiple countries and continued investment in networked surveillance capacity (AMRSNET) [71]. Key drivers include: antibiotic dispensing without prescription, varying infection control standards, sanitation quality, livestock antibiotic use, healthcare funding, and surveillance completeness [72].

A recent hospital-based study from the Middle East confirmed that imipenem-resistant *P. aeruginosa* infection rates in the region are alarmingly high, consistent with global surveillance data [73]. Encouragingly, the same study showed that a structured set of infection control measures, including regular surveillance and stricter isolation protocols, cut those rates by nearly 60%, proving that such resistance, while serious, can be meaningfully controlled with the right institutional response to 76% over a single calendar year [73].

### 3.4. Pathogen–Drug Combinations of Greatest Concern (2025 View)

The Enterobacterales (notably *E. coli* and *K. pneumoniae*) with resistance to 3GC and carbapenems dominate the global clinical signal, narrowing empiric choices for urinary tract and bloodstream infections. GLASS 2025 and contemporaneous syntheses highlight escalating challenges with GNB, including carbapenem-resistant *A. baumannii* and fluoroquinolone-resistant *Salmonella* spp. (e.g., *S. typhi*, *S. paratyphi*) in several regions [3]. These trends align with the WHO 2024/2025 priority pathogen updates that continue to flag critical-priority GNB (for clinical relevance, see Section 4 for pipelines and non-traditional interventions).

### 3.5. Surveillance Ecosystem Upgrades in 2025

A notable 2025 systems change is the splitting of the U.S. National Healthcare Safety Network, Antimicrobial Use and Resistance (NHSN AUR) measure into two separate measures, Antimicrobial Use and Antimicrobial Resistance, for the 2025 EHR reporting period, as reflected in Centres for Medicare & Medicaid Services (CMS) and CDC updates and NHSN office-hours materials. The change clarifies reporting expectations and may improve data quality and stewardship feedback loops at facility level [71]. Globally, GLASS participation rose in recently, though WHO notes variable data completeness, particularly in regions with constrained laboratory infrastructure—a core target for international investment [3].

### 3.6. Interpreting the Numbers: Caveats and Modelled Synthesis

While the “one in six” headline is robust, several caveats apply for scientific interpretation:

1. Case-mix and testing intensity differ across countries and care levels; under-testing can bias prevalence downward or upward depending on sampling frames.
2. Resistance definitions and Antimicrobial Susceptibility Testing (AST) breakpoints are not fully harmonized in all contributing labs.
3. Denominator effects (e.g., shifts in care-seeking or culture practices post-COVID) may influence year-over-year trends.
4. Modelled burden estimates (mortality/DALYs) depend on counterfactual assumptions about treatment/failure risks.

Even with these caveats, triangulating GLASS prevalence, regional surveillance, and modelled burden supports the conclusion that AMR prevalence increased globally from 2018–2023, with highest proportions in South-East Asia/Eastern Mediterranean, substantial challenges in Africa, and continued but comparatively lower levels in Europe/Western Pacific (Table 1).

Interpreting global AMR trends requires a nuanced approach, as several technical and geographic disparities can significantly skew the accuracy and comparability of surveillance data [3,11,74]:

- Infection types and pathogen inclusion criteria vary by country
- Uneven coverage creates systematic bias (high-income overrepresented)
- AST standards: 68% use EUCAST, 32% CLSI, cross-comparison limited
- Post-COVID denominator shifts (reduced routine testing) inflate resistance percentages
- DALY/mortality estimates rely on counterfactual assumptions; uncertainty ranges ±20–30%
- NHSN AUR decoupling disrupted historical trends; transitional data require caution

The description of Africa is overly generalized and would benefit from country-level differentiation.

### 3.7. Clinical Implications in 2025

Clinicians face shrinking empiric windows, defined as the period during which first-line therapy is likely effective before susceptibility results return, currently 12–48 h [75], for severe community and hospital infections, particularly Urinary Tract Infections (UTIs) and Bloodstream Infections (BSIs) caused by Extended-Spectrum Beta-Lactamase-producing Enterobacterales (ESBL-E) and carbapenem-resistant Non-fermenting GNB [76,77]. The mismatch between rising resistance and limited access to new or last-resort agents (and to rapid diagnostics) remains acute in high-burden regions [3,78]. These pressures justify the Antimicrobial Use/Antimicrobial Resistance decoupling (U.S.) for sharper stewardship feedback and underscore the WHO call for equitable access to effective antibiotics and diagnostics [3].

## 4. Antibiotic Innovation

**Figure 3:**
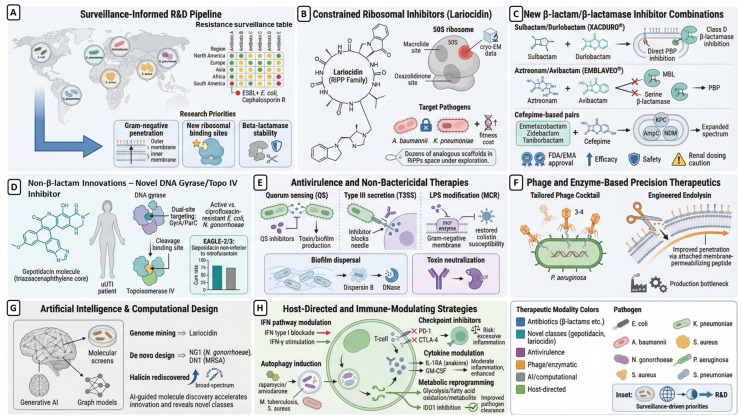
Overview of next-generation antibacterial innovation strategies integrating surveillance-informed R&D, novel target discovery, and precision therapeutics. The schematic highlights: (**A**) global resistance surveillance guiding research priorities (Gram-negative penetration, new ribosomal binding sites, β-lactamase stability); (**B**) constrained ribosomal inhibitors (e.g., lariocidin) targeting the 50S subunit; (**C**) new β-lactam/β-lactamase inhibitor combinations; (**D**) non-β-lactam innovations such as DNA gyrase/topoisomerase IV inhibitors; (**E**) antivirulence approaches including quorum sensing inhibition, T3SS blockade, LPS modification targeting, biofilm dispersal, and toxin neutralization; (**F**) phage and enzyme-based precision therapeutics; (**G**) artificial intelligence–driven molecular discovery and (**H**) host-directed immunomodulatory strategies. Therapeutic modalities and key target pathogens are indicated.

Despite the persistent stagnation that characterized the early 2020s, the last years have seen a modest reawakening of antibiotic innovation. This shift did not occur spontaneously: it has been propelled by parallel developments in genomic discovery platforms, structure-guided drug design, and the maturation of AI-based molecule screening systems. Industry participation remains thin, yet a growing number of academic–industry consortia have begun to fill this void, accelerating early-stage discovery of compounds directed at the most urgent threats identified in global surveillance [3]. As resistance rates continue to rise across Enterobacterales and non-fermenters (see Section 3), research priorities have pivoted decisively toward agents with improved Gram-negative cell wall penetration, novel ribosomal binding sites, and β-lactamase stability [79] (Figure 3).

These shifts in scientific priorities closely mirror the resistance patterns captured through international monitoring systems, particularly the pathogen–antibiotic combinations most under pressure between 2016 and 2023, as summarized in Table 2. The concentration of surveillance efforts on carbapenems, advanced cephalosporins, and key fluoroquinolones underscores the escalating threat posed by multidrug-resistant GNB and directly informs the strategic emphasis of the discovery programs discussed below (Figure 3).

The renewed momentum is nonetheless fragile. Economic analyses published, consistently reaffirm that antibiotic R&D remains financially unattractive without pull incentives or subscription-style reimbursement models [80]. Thus, the scientific progress summarized in this section must be interpreted within the broader constraints of global access, stewardship, and policy, issues revisited in subsequent sections.

### 4.1. Lariocidin and the Rise of Constrained Ribosomal Inhibitors

One of the most consequential findings is the characterization of lariocidin, a structurally constrained lasso peptide isolated from *Brevibacillus* sp. and described in Nature in early 2025 [12]. Lariocidin belongs to the expanding family of Ribosomally synthesized and Post-translationally modified Peptide (RiPP)-derived antibiotics whose stability and protease resistance arise from their unique macrolactam “lasso” conformation [12] (Figure 3).

Unlike classical ribosomal inhibitors, lariocidin binds a previously uncharacterized pocket on the 50S subunit, distinct from macrolide and oxazolidinone sites. Structural work using Cryo-Electron Microscopy (cryo-EM) has shown that this binding event disrupts peptidyl-transferase center dynamics without inducing the conformational rearrangements typical of existing drugs [12]. Functionally, the compound exhibits potent activity against CR *A. baumannii* and *K. pneumoniae*, pathogens that drive some of the highest mortality signals in GLASS 2025 [3,81]. Laboratory evolution assays indicate a comparatively high barrier to resistance, likely because mutations in the targeted pocket incur substantial fitness penalties [82].

Lariocidin has therefore drawn intense interest not simply as a lead molecule but as a representative of a much broader, and previously underexplored, chemical space. Computational mining of RiPP biosynthetic gene clusters suggests dozens of analogous scaffolds with pharmaceutical potential, pointing toward a new generation of ribosomal inhibitors [12] (Figure 3).

Quantitatively, lariocidin MIC_90_ ranges 0.5–4 µg/mL against *A. baumannii* and *K. pneumoniae*, comparable to colistin but with narrower therapeutic index [12]. Resistance emergence frequency ~10^−9^ (versus ~10^−8^ for rifampin). Pharmacodynamic studies indicate time-dependent killing [12]; clinical translation remains early-stage (Phase I pending).

### 4.2. New Inhibitors

Recent progress in antimicrobial development has generated several promising small-molecule agents and β-lactam/β-lactamase inhibitor combinations aimed at countering the rising prevalence of Extended-Spectrum β-Lactamase (ESBL)-producing Enterobacterales and metallo-β-lactamases (Section 3). Among these innovations, sulbactam/durlobactam (XACDURO^®^, Waltham, MA, USA) represents a major achievement, revitalizing the long-standing β-lactamase inhibitor sulbactam, already known for intrinsic activity against *Acinetobacter* [83,84] through its pairing with durlobactam, a next-generation diazabicyclooctane inhibitor specifically engineered to protect sulbactam from degradation by modern β-lactamase-producing *A. baumannii* [85,86,87]. This complementary mechanism, whereby sulbactam directly binds PBPs while durlobactam neutralizes a broad spectrum of serine β-lactamases, notably Class D enzymes [85,86,88], restores potent activity against carbapenem-resistant *A. baumannii*. Clinical validation came from randomized trials in severe infections such as Hospital-Acquired Pneumonia/Ventilator-Associated Pneumonia HAP/VAP, showing improved clinical and microbiologic outcomes compared with best available therapy [89,90]. With safety concerns limited primarily to infusion reactions, hypersensitivity, and renal dosing considerations [3,90], the Food and Drug Administration (FDA) approved XACDURO^®^ in May 2023 for infections caused by the *A. baumannii–calcoaceticus* complex [84,86,89].

A similar rational design underlies aztreonam/avibactam (EMBLAVEO^®^): aztreonam is naturally stable to MBLs but vulnerable to serine β-lactamases [91], while avibactam inhibits serine enzymes, *Klebsiella pneumoniae* carbapenemase (KPC), AmpC, and Extended-Spectrum β-Lactamases (ESBLs), yet lacks MBL activity [92]. Their combination therefore provides dual protection, enabling aztreonam to retain activity against organisms that co-produce MBLs and additional β-lactamases [92,93]. This mechanistic logic was corroborated by phase II/III programs and real-world compassionate-use data (NCT03329092; NCT03580044), which demonstrated strong clinical and microbiologic responses in cIAI, cUTI, and HAP/VAP due to difficult-to-treat GNB, including MBL producers [92,93]. With a favourable safety profile and a need for stewardship emphasized in product labelling, the EMA authorized EMBLAVEO^®^ in 2024, followed by FDA approval in early 2025 [92,94].

Parallel development efforts revitalized cefepime through combination strategies. Cefepime/enmetazobactam (EXBLIFEP^®^, London, UK.), pairing a 4th-generation cephalosporin with a novel sulfone-based β-lactamase inhibitor, expands activity against ESBL- and some carbapenemase-producing Enterobacterales [95,96]. Global phase II/III trials demonstrated non-inferiority to meropenem and favourable eradication and cure rates in resistant subgroups [96,97], supporting regulatory approval in 2024. Its safety profile aligns with that of cefepime, particularly neurotoxicity risks in renal impairment, without unexpected effects from enmetazobactam [97,98]. Additional agents include cefepime–zidebactam, which combines β-lactamase inhibition with high-affinity PBP2 binding; phase III trials (NCT04979806) completed in 2024–2025 show promise against KPC- and AmpC-producing Enterobacterales [97,98,99]. Cefepime–taniborbactam, targeting both serine and metallo-β-lactamases including NDM [100], remains under extended regulatory review due to the complexity of demonstrating consistent efficacy across evolving MβL variants [101].

Beyond β-lactams, gepotidacin (BLUJEPA^®^, London, UK) offers a first-in-class triazaacenaphthylene scaffold, representing the first new chemical class of oral antibiotics for uncomplicated urinary tract infections (uUTIs) in over two decades [102,103]. Unlike fluoroquinolones, which target a single binding site on DNA gyrase and topoisomerase IV, gepotidacin exhibits a unique dual-targeting mechanism: it binds with equal affinity to both enzymes at a distinct site (the GyrA/ParC cleavage junction), stabilizing double-stranded DNA breaks and blocking bacterial DNA replication [102,103]. This distinct binding mode circumvents common fluoroquinolone resistance mutations (e.g., in *gyrA* or *parC*), explaining its retained activity against ciprofloxacin-resistant *E. coli* and *N. gonorrhoeae* clinical isolates.

The clinical development program was robust. In the pivotal phase 3 trials, EAGLE-2 and EAGLE-3, gepotidacin (1500 mg twice daily for 5 days) was compared to nitrofurantoin (100 mg twice daily for 5 days) in over 3000 women with uncomplicated UTIs. Both trials met the primary endpoint of therapeutic non-inferiority, with gepotidacin demonstrating composite cure rates (clinical + microbiological response) of 62.5% and 61.7%, respectively, compared to 59.6% and 57.9% for nitrofurantoin [104,105]. Notably, in the EAGLE-3 trial, gepotidacin showed superior efficacy in a prespecified subgroup analysis against pathogens resistant to first-line antibiotics [105]. The safety profile was favourable, with most adverse events being mild to moderate gastrointestinal symptoms (i.e., diarrhoea, nausea), and no evidence of tendon toxicity or QT prolongation observed with fluoroquinolones.

Based on these results, the FDA approved gepotidacin in March 2025 for the treatment of adult women with uncomplicated UTIs caused by susceptible isolates of *E. coli*, *K. pneumoniae*, and *S. saprophyticus* [105]. Regulatory submissions to other agencies (EMA, UK MHRA) are pending. Beyond uUTIs, gepotidacin is under investigation for uncomplicated urogenital gonorrhoea (phase 3 EAGLE-1 trial), where its oral formulation and activity against resistant *N. gonorrhoeae* could offer a much-needed alternative to injectable ceftriaxone [106]. A paediatric formulation is also in development (NCT07371429).

Quantitatively, the antibacterial development pipeline remains critically insufficient despite recent advances. According to the World Health Organization 2025 antibacterial pipeline analysis, approximately 90 antibacterial agents are currently in preclinical or clinical development; however, only a limited number target WHO priority multidrug-resistant pathogens, and even fewer are considered truly innovative [107]. WHO emphasized in 2026 the urgent need for “new antibacterial agents that are innovative, affordable, and accessible to all those who need them,” particularly against severe multidrug-resistant Gram-negative infections caused by carbapenem-resistant *Acinetobacter baumannii*, *Pseudomonas aeruginosa*, and Enterobacterales [108]. Within this context, lariocidin has emerged as a promising candidate due to its novel mechanism of action [109]. The compound demonstrated potent antibacterial activity against multidrug-resistant Gram-negative pathogens, including carbapenem-resistant *A. baumannii* and *Klebsiella pneumoniae*, with low MIC values reported across susceptible strains and a very low frequency of spontaneous resistance emergence (<10^−9^) [109]. Mechanistically, lariocidin binds to a previously unexploited site on the 30S ribosomal subunit, thereby inhibiting bacterial protein synthesis through a distinct translational interference mechanism. In vivo efficacy was confirmed in murine models of *A. baumannii* infection, while minimal mammalian cytotoxicity was observed in preclinical evaluations [110]. Nevertheless, despite its strong therapeutic potential, lariocidin remains at the preclinical stage, highlighting the persistent gap between antibacterial discovery and clinical translation.

### 4.3. Antivirulence and Non-Bactericidal Modalities

Parallel to classical small-molecule discovery, research broadened into antivirulence therapies, agents that disable pathogenic mechanisms while exerting weaker selective pressure for resistance. Current pipelines include inhibitors of Quorum-Sensing (QS) circuits [111], Type III Secretion Systems (T3SS) [112], and lipopolysaccharide (LPS) modification pathways [113,114].

Of particular relevance to global surveillance trends is the effort to counteract colistin resistance mediated by *mcr* genes [115,116]. Several groups have identified compounds that inhibit phosphoethanolamine transferases, partially restoring colistin susceptibility in *mcr*-positive Enterobacterales [117,118,119]. Although these approaches remain preclinical, they exemplify a shift toward therapeutics aimed at resistance reversal rather than direct bactericidal activity.

Parallel to classical small-molecule discovery, research has broadened into antivirulence therapies, an approach that disables bacterial pathogenic mechanisms without directly killing the pathogen. By disarming bacteria rather than exerting bactericidal pressure, these strategies aim to perturb infection establishment and progression while applying weaker selective pressure for resistance development, potentially preserving the host microbiota [120].

Several virulence pathways are under active investigation as therapeutic targets. QS inhibitors disrupt bacterial cell-to-cell communication, preventing the coordinated expression of virulence factors such as toxins, proteases, and biofilm formation. In *P. aeruginosa*, for instance, inhibitors of the LasR and RhlR QS systems have shown promise in preclinical models, attenuating virulence and enhancing bacterial clearance by host immunity [121]. T3SS inhibitors block the injection of effector proteins directly into host cells, a critical virulence mechanism for pathogens like *P. aeruginosa*, *Salmonella*, and enteropathogenic *E. coli*. Small molecules targeting the T3SS apparatus or its ATPase have demonstrated efficacy in reducing tissue damage and inflammation in animal models [121].

Another major focus is the inhibition of LPS modification pathways, particularly those conferring resistance to last-resort polymyxins [122]. The MCR enzymes (phosphoethanolamine transferases) responsible for colistin resistance are attractive targets: several research groups have identified small-molecule inhibitors that block MCR activity, partially restoring colistin susceptibility in *mcr*-positive Enterobacterales in vitro and in vivo [122]. While these remain preclinical results, they exemplify a “resistance reversal” strategy that could extend the clinical lifespan of existing antibiotics.

Biofilm dispersal agents represent another antivirulence avenue. Enzymes such as dispersin B (targeting poly-N-acetylglucosamine in *S. aureus* and *E. coli* biofilms) and DNases that degrade extracellular DNA in the biofilm matrix have been shown to enhance antibiotic penetration and efficacy in experimental models [123]. In contrast to these matrix-disrupting enzymes, which physically dismantle biofilm structures, toxin neutralization strategies operate through a different mechanism: monoclonal antibodies or small-molecule inhibitors are being developed against key virulence factors such as *C. difficile* toxins TcdA/TcdB and *S. aureus* alpha-hemolysin, with some candidates advancing into clinical trials [124].

Nevertheless, it is important to note that long-term effectiveness data are absent; antivirulence agents may still select for resistance via alternative pathways (e.g., virulence factor upregulation or compensatory mutations). No clinical studies have yet demonstrated durability beyond 6 months.

### 4.4. Phage-Based and Enzyme-Based Precision Therapeutics

Phage therapy continued its transition from experimental to semi-formalized clinical evaluation. Multiple compassionate-use successes prompted controlled trials investigating tailored phage cocktails for *P. aeruginosa* and *A. baumannii* infections [125,126,127,128]. In parallel, engineered endolysins, peptidoglycan hydrolases linked to membrane-permeabilizing peptides, have demonstrated activity against GNB previously considered inaccessible to lysins [129,130,131]. These biologics provide advantages such as specificity and a reduced likelihood of off-target microbiome disruption, though production scalability remains a major barrier.

Key barriers remain: narrow host range requiring personalized matching, bacterial phage resistance, regulatory uncertainty (no standardized approval pathway), manufacturing reproducibility, and immunogenicity with repeated dosing. In fact, phage therapy remains limited by regulatory fragmentation: Belgium and the USA allow compassionate use, but no phage product has received full FDA/EMA approval for systemic bacterial infections. Controlled trials (e.g., PhagoBurn, NCT04682964) showed mixed efficacy and no superiority over standard antibiotics in some endpoints [132]. Safety concerns include immunogenicity, endotoxin release, and horizontal transfer of resistance genes. Most published evidence comes from compassionate use case series, lacking randomization or blinding. Until phase III data emerge, phage therapy should be viewed as an adjunctive, not standalone, strategy.

### 4.5. Artificial Intelligence and Computational Design

AI has rapidly evolved from a screening adjunct to a central driver of early-stage antibiotic discovery. Deep neural networks trained on antimicrobial activity datasets now predict membrane permeation, efflux susceptibility, and toxicity with unprecedented accuracy (Figure 3). Generative models have produced novel peptide antibiotics with enhanced serum stability, while graph-based models have identified small molecules structurally divergent from existing classes. In multiple cases, computational predictions have outpaced traditional wet-lab screening, significantly reducing discovery timescales [133,134,135].

Importantly, AI-guided genome mining was instrumental in the identification of lariocidin, underscoring how computational approaches can reveal cryptic natural products previously invisible to classical cultivation-based methods [109]. Furthermore, over the past two years, generative AI has accelerated antibiotic discovery, with a Massachusetts Institute of Technology (MIT)-led study in *Cell* describing the design of millions of hypothetical compounds and identification of two leads, NG1, active against drug-resistant *Neisseria gonorrhoeae*, and DN1, effective against methicillin-resistant *Staphylococcus aureus* (MRSA) in mouse models, generated via fragment-based and unconstrained de novo AI models exploring novel chemical space [136]. Early AI-based antimicrobial discovery predates this work, with deep learning-identified halicin rediscovered as a broad-spectrum antibiotic capable of killing multiple drug-resistant bacteria, highlighting the potential of computational approaches for identifying non-traditional mechanisms of action.

Despite these remarkable advances, a substantial translational gap remains between AI-driven discovery and clinical application. The vast majority of AI-identified antibiotic candidates, including halicin and many generative model outputs, have been validated only in preclinical in vitro or murine models, with very few progressing to human trials [135]. Key barriers include limited assessment of safety profiles (e.g., cytotoxicity, hemolytic activity, off-target effects), unpredictable pharmacokinetics and bioavailability in humans, and a lack of standardized pipelines for the clinical validation of computationally derived molecules [137,138]. Furthermore, the “black box” nature of some deep learning models complicates mechanistic understanding and regulatory acceptance. Addressing these gaps will require integrated efforts between computational biologists, medicinal chemists, and clinicians to establish robust preclinical-to-clinical translation frameworks specifically tailored for AI-generated antibiotics [138].

### 4.6. Host-Directed and Immune-Modulating Strategies

A smaller but rapidly growing part of the pipeline targets host pathways rather than bacterial viability. These host-directed therapies (HDTs) aim to enhance the innate and adaptive immune response, modulate inflammation, or create a cellular environment unfavourable to pathogen persistence [139,140]. By acting on host factors rather than bacterial targets, HDTs theoretically apply less selective pressure for resistance and may be effective across multiple pathogens sharing similar intracellular niches [139,140].

Several strategies are under active investigation. Modulation of interferon responses has emerged as a promising approach to enhance intracellular bacterial clearance. Type I interferons, while essential for antiviral defence, can be detrimental during bacterial infections by suppressing protective immune responses [141]. In preclinical models of tuberculosis, blockade of the type I interferon pathway using neutralizing antibodies or small-molecule inhibitors of the Interferon-Alpha/Beta (IFNAR) receptor improved bacterial control and reduced pathology [142]. Conversely, stimulation of type II interferon (IFN-γ) with recombinant IFN-γ has been used as adjunctive therapy in chronic granulomatous disease and nontuberculous mycobacterial infections, though its use remains limited by toxicity and variable efficacy (Figure 3).

Pharmacologic activation of autophagy, a cellular degradation pathway that eliminates intracellular pathogens, represents another major HDT avenue [143]. Autophagy inducers such as rapamycin (mTOR inhibitor) and its analogues have shown promise in preclinical models of tuberculosis, salmonellosis, and *S. aureus* infections, enhancing bacterial clearance and reducing inflammatory damage [144]. More recently, the antiarrhythmic drug amiodarone was identified through a phenotypic screen as a potent autophagy enhancer capable of restricting mycobacterial growth in macrophages and zebrafish models, demonstrating the potential for repurposing approved drugs for host-directed effects [139]. Clinical trials are now evaluating the autophagy inducer everolimus as adjunctive therapy in pulmonary tuberculosis (NCT number: NCT02968927).

Immune checkpoint inhibitors, best known in oncology, are being explored for their potential to reverse immune exhaustion in chronic infections [145]. Programmed cell death protein 1 (PD-1) and cytotoxic T-lymphocyte-associated protein 4 (CTLA-4) blockade has been shown to enhance T-cell responses against *M. tuberculosis* and *S. aureus* in animal models, though concerns about excessive inflammation and immune-related adverse events remain significant [146].

Cytokine modulation strategies aim to fine-tune the inflammatory response to achieve optimal bacterial clearance while minimizing tissue damage [147]. Interleukin-1 receptor antagonists (e.g., anakinra) have been evaluated in septic shock and severe pneumonia to temper hyperinflammation, with mixed results [148]. Conversely, administration of granulocyte-macrophage colony-stimulating factor (GM-CSF) has been used to enhance neutrophil and macrophage function in immunocompromised hosts with refractory infections [149].

Metabolic reprogramming of immune cells is an emerging frontier. Pathogens often exploit host metabolic pathways to establish persistent infection [150]. Small molecules that modulate glycolysis, fatty acid oxidation, or amino acid metabolism in macrophages and T cells are being explored for their ability to create a hostile metabolic environment for intracellular pathogens [151]. For example, inhibition of the enzyme IDO1 (indoleamine 2,3-dioxygenase), which depletes tryptophan and suppresses T-cell responses, has shown benefit in preclinical models of tuberculosis and sepsis [152].

An emerging complement to classical host-directed therapy involves the microbiome. The resident microbial community influences infection outcomes through colonization resistance (competing with pathogens for nutrients and niches), immune signalling modulation (e.g., short-chain fatty acids that enhance regulatory T cell function), and direct effects on immunotherapy efficacy [153,154]. Disruption of the gut microbiota by broad-spectrum antibiotics increases susceptibility to infections such as *Clostridioides difficile* [155]. Accordingly, microbiome-targeted interventions, including probiotics, faecal microbiota transplantation (FMT), and microbiome-sparing antibiotics, are gaining traction as adjunctive host-directed strategies, though standardization and safety remain challenges [156].

While unlikely to replace antibiotics, these host-directed agents may function as adjunct therapies that reduce relapse rates, shorten treatment durations, or improve outcomes in hard-to-treat infections, particularly those involving intracellular pathogens (tuberculosis, salmonellosis, listeriosis) or biofilm-associated chronic infections where conventional antibiotics often fail to eradicate persistent reservoirs.

## 5. Are We Finally Learning to Run Faster? Strategic Priorities Beyond 2025

**Figure 4:**
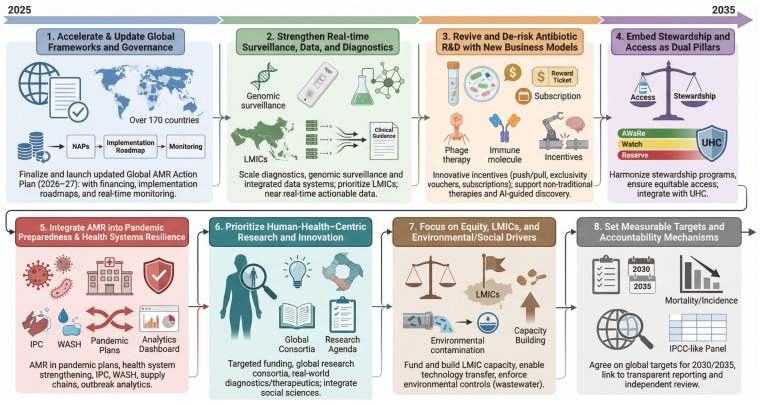
Strategic global roadmap (2025–2035) to combat antimicrobial resistance (AMR), outlining eight coordinated pillars: (1) accelerating and updating global governance frameworks; (2) strengthening real-time genomic surveillance, diagnostics, and integrated data systems—particularly in LMICs; (3) revitalizing and de-risking antibiotic R&D through innovative business models and incentives; (4) embedding stewardship and equitable access within Universal Health Coverage (UHC); (5) integrating AMR into pandemic preparedness and health system resilience (IPC, WASH, analytics); (6) prioritizing human-health–centric research and global innovation consortia; (7) advancing equity, capacity building, and environmental controls; and (8) establishing measurable 2030/2035 targets with transparent accountability mechanisms to reduce AMR-related mortality and incidence.

In recent years, the discourse around AMR has shifted from recognition of the problem toward action frameworks and strategic priority-setting. While progress has been made, to truly outpace the accelerating emergence of resistant pathogens requires not just incremental improvements but a marked increase in speed, coordination and innovation. As we cross the midpoint of the 2020s, several strategic priorities stand out if we are to run faster and meaningfully reverse or mitigate the projected trajectory of resistance (Figure 4).

### 5.1. Accelerate and Update Global Frameworks & Governance

WHO in May 2025 announced that the 78th World Health Assembly approved a decision to update the Global Action Plan on Antimicrobial Resistance (GAP-AMR) (2015) for the next decade, emphasizing multisectoral “One Health” integration and stronger implementation support for countries [157]. The justification for this update is grounded in the widening gap between policy commitments and realized outcomes: though over 170 countries have NAPs (National Action Plans) aligned to GAP-AMR, many lack full implementation, finance, monitoring and evaluation systems [158].

Strategic priority #1 thus is: finalize and launch the updated global framework by 2026–2027, with built-in financing mechanisms, implementation roadmaps, and real-time monitoring dashboards.

### 5.2. Strengthen Real-Time Surveillance, Data, Diagnostics

The newly released Global Antibiotic Resistance Surveillance Report 2025 (based on over 23 million infections from 104 countries) demonstrates both how far surveillance has progressed and how much remains to be done [158]. Key gaps include: delayed data feeds, lack of diagnostics in low-resource settings, limited pathogen coverage, and insufficient linkage between surveillance and clinical decision-making.

Strategic priority #2: Invest in and scale rapid diagnostics, genomic surveillance, integrated data systems, particularly in Low- and Middle-Income Countries (LMICs). This includes deploying point-of-care AMR diagnostics, strengthening laboratory networks, and creating interoperable data that inform stewardship in near real time. Furthermore, addressing the diagnostic gap requires technologies that deliver actionable results within clinically relevant timeframes. Recent advances in rapid antimicrobial susceptibility testing (AST) now enable single-bacterial response measurement within 30 min using fluorescent or functional probes [159,160]. Complementary platforms targeting cell-activity-related molecular determinants offer on-site feasibility [161]. When deployed at point-of-care, such rapid ASTs can guide empiric therapy, reduce inappropriate antibiotic use, and strengthen real-time stewardship, directly addressing the surveillance priorities highlighted by GLASS 2025.

### 5.3. Revive and De-Risk Antibiotic R&D with New Business Models

One of the most persistent bottlenecks remains the antibiotic pipeline: despite scientific advances, economic incentives are often misaligned (low return on investment, short durations of use, high risk). The World Economic Forum’s Davos Compact 2025 calls for “sustainable antimicrobial R&D ecosystems” that combine push funding, pull incentives, appropriate access and stewardship [162].

Strategic priority #3: Implement innovative funding models—e.g., market-entry rewards, transferable exclusivity vouchers, subscription models—to stimulate early- and late-stage antibiotic development. In parallel, non-traditional therapies (phages, immune modulation, adjuvants) and AI-guided discovery should receive scaled support.

### 5.4. Embed Stewardship and Access as Dual Pillars

A key tension continues: ensuring access to effective antibiotics for all, while conserving their utility through strong stewardship. The 2025 WHO fact-sheet emphasizes that misuse across human, animal, plant and environmental sectors remains the driver of resistance [162,163].

Strategic priority #4: Implement globally harmonized stewardship programs aligned with the Access, Watch, Reserve (AWaRe) classification, and ensure equitable access to quality assured antibiotics and diagnostics. Integration of stewardship into Universal Health Coverage (UHC) financing is also essential. AWaRe implementation faces substantial challenges, particularly in LMICs. Supply security remains a key barrier: only 29 of 39 AWaRe-listed antibiotics are available in some countries, with no Reserve group agents accessible [164]. Diagnostic dependence further complicates appropriate Watch/Reserve use, as many LMIC facilities lack laboratory capacity to identify resistant infections requiring these agents [164]. Without rapid diagnostics, even well-intentioned AWaRe adherence may default to empirical broad-spectrum prescribing, undermining stewardship goals.

### 5.5. Integrate AMR into Pandemic-Preparedness and Health-System Resilience

COVID-19 exposed a deadly synergy: fragile health systems amplify threats like AMR. Integrating AMR containment into UHC and pandemic preparedness is now a non-negotiable pillar of health security [165,166].

Strategic priority #5: Embed AMR monitoring, response and innovation into national pandemic-preparedness plans and health-systems strengthening efforts. This means cross-linking AMR with Infection Prevention & Control (IPC), Water, Sanitation, Hygiene (WASH) infrastructure, supply-chain resilience, and outbreak analytics.

### 5.6. Prioritize Human-Health–Centric Research and Innovation

While the One Health approach remains vital, the human-health domain warrants renewed focus: the WHO Global Research Agenda for Antimicrobial Resistance in Human Health has identified 40 research priorities by 2030 covering prevention, diagnosis, treatment and policy translation [167].

Strategic priority #6: Allocate targeted funding and build global consortia around the research agenda—especially for rapid diagnostic validation, therapeutic adversarial resistance design, real-world stewardship implementation science. Cross-disciplinary work (e.g., social sciences) is also key, as a recent 2025 virtual dialogue emphasized [168].

### 5.7. Focus on Equity, LMICs, and Environmental/Social Drivers

AMR is both a medical and a social challenge. Structural issues—limited diagnostics in LMICs, lack of sanitation, antimicrobial contamination of environments—underlie persistent transmission and selection of resistance. Ahmed et al. emphasize that this is a “tragedy of the commons” problem requiring global shared responsibility [169].

Strategic priority #7: Ensure LMIC-tailored funding, capacity building, technology transfer, and environmental controls (e.g., antibiotic residues in wastewater). This is foundational for sustainable AMR control.

### 5.8. Set Measurable Targets and Accountability Mechanisms

At the United Nations General Assembly High-Level Meeting on AMR, world leaders committed to a 10% reduction in AMR-associated deaths by 2030 [170,171]. However, without robust accountability, monitoring and enforcement mechanisms, such targets risk becoming aspirational.

Strategic priority #8: Agree on global targets for 2030/2035 (e.g., incidence, mortality, pipeline delivery, stewardship coverage), linked to transparent reporting and independent review (similar to the Intergovernmental Panel on Climate Change (IPCC) model for climate).

## 6. Conclusions

The fight against antimicrobial resistance is a race against bacterial evolution, one that humanity is not yet winning. While scientific breakthroughs in drug discovery, diagnostics, and alternative therapies offer a renewed sense of hope, they are insufficient alone. Turning the tide requires a durable, globally coordinated strategy that marries innovation with implementation. Strengthening health systems, ensuring equitable access to existing and new antibiotics, and embedding robust stewardship into the fabric of healthcare are no longer optional but essential. The future of modern medicine depends on our collective ability to outpace adaptation with smarter, faster, and more unified action.

## Figures and Tables

**Table 1:** Global AMR situation in 2025: synthesized view from GLASS and regional systems [3,11,74]. Non-participants (GLASS 2023): Africa (20/47 countries), Eastern Mediterranean (5/21), South-East Asia (1/11), Europe (22/53), Western Pacific (17/37), Americas (28/35).

Region	Approx. Proportion of Lab-Confirmed Infections Resistant (All-Pathogen Aggregate)	Notable High-Concern Combinations	Surveillance Notes
South-East Asia	~1 in 3	3GC-R *E. coli/K. pneumoniae*; FQ-R *Salmonella*; CR-*A. baumannii*	GLASS 2025
Eastern Mediterranean	~1 in 3	3GC-R Enterobacterales; CR-*Acinetobacter*/*Pseudomonas* (Non-fermenting GNB)	GLASS 2025
Africa	Heterogeneous; very high in several settings; >70% resistance to first-line agents for some BSIs reported	3GC-R Enterobacterales; CR-*A. baumannii*	GLASS 2025; Africa CDC briefs/studies.
Europe	~1 in 10 (aggregate), but rising signals for key pathogens	3GC-R *E. coli/K. pneumoniae*; CR-*Acinetobacter* in select countries	EARS-Net/CAESAR 2023 data; GLASS regional summary
Western Pacific	~1 in 11	Enterobacterales (3GC-R); variable MRSA	GLASS 2025 summaries/coverage
Americas	Mixed; pockets of high resistance in hospital networks	ESBL-E; CR-Enterobacterales; rising *Acinetobacter* signals	GLASS 2025; NHSN AUR upgrade context (data quality)

Abbreviations: 3GC-R: 3rd-generation cephalosporin resistant; FQ-R = fluoroquinolone resistant; CR = carbapenem-resistant; MRSA: Methicillin Resistant *Staphylococcus aureus*; ESBL-E: Extended-spectrum beta-lactamase-producing Enterobacterales; GLASS: Global Antimicrobial Resistance Surveillance System; NHSN AUR: U.S. National Healthcare Safety Network, Antimicrobial Use and Resistance; EARS-Net: European Antimicrobial Resistance Surveillance Network.

**Table 2:** Infection type, bacterial pathogen and antibiotic combinations under surveillance between 2016 and 2023 and included in Global antibiotic resistance surveillance report 2025 [3].

	*Acinetobacter* spp.	*E. coli*	*K. pneumoniae*	*N. gonorrhoeae*	*Salmonella* spp. ^2^	*Shigella* spp.	*S. aureus*	*S. pneumoniae*
Aminoglycosides								
Amikacin	BSI							
Gentamicin	BSI			STI				
Spectinomycin				STI				
Carbapenems								
Doripenem	BSI	BSI UTI	BSI UTI		BSI GTI			
Ertapenem		BSI UTI	BSI UTI		BSI GTI			
Imipenem	BSI ^1^	BSI ^1^ UTI ^1^	BSI ^1^ UTI ^1^		BSI GTI			
Meropenem	BSI	BSI UTI	BSI UTI		BSI GTI			
2nd-generation Cephalosporins								
Cefoxitin ^3^							BSI	
3rd-generation Cephalosporins								
Ceftriaxone		BSI UTI	BSI UTI		BSI GTI	GTI		BSI
Ceftazidime		BSI UTI	BSI UTI		BSI GTI	GTI		
Cefotaxime		BSI ^1^ UTI ^1^	BSI ^1^ UTI ^1^		BSI GTI	GTI		BSI
Cefixime				STI				
4th-generation Cephalosporins								
Cefepime		BSI UTI	BSI UTI					
Fluoroquinolones								
Ciprofloxacin		BSI UTI	BSI UTI	STI	BSI ^1^ GTI ^1^	GTI ^1^		
Levofloxacin		BSI UTI	BSI UTI		BSI GTI	GTI		
Macrolides								
Azithromycin				STI		GTI		
Penicillins								
Oxacillin ^3,4^							BSI	BSI
Penicillin G								BSI ^1^
Polymyxins								
Colistin	BSI	BSI UTI	BSI UTI					
Sulfonamides and trimethoprim								
Co-trimoxazole		BSI UTI	BSI UTI					BSI
Tetracyclines								
Minocycline	BSI							
Tigecycline	BSI							

^1^: Marks selected pathogen–antibiotic combinations for which national percentage resistance estimates and 2018–2023 resistance trends are reported. These are also presented for MRSA and 3GC-R *E. coli*. ^2^: *S. Typhi* and *S. Paratyphi* are not included in this report. ^3^: Both cefoxitin and oxacillin are penicillinase-stable beta-lactams. The Clinical and Laboratory Standards Institute (CLSI) and the European Committee on Antimicrobial Susceptibility Testing (EUCAST) recommend use of cefoxitin instead of oxacillin for disc diffusion testing to determine methicillin resistance in *S. aureus*. Cefoxitin is used as a surrogate for assessing susceptibility to oxacillin (as well as methicillin and nafcillin). As countries reported results for either or both agents, in this report, MRSA is calculated from AST results for oxacillin and/or cefoxitin. ^4^: Oxacillin AST can be used as a surrogate for benzylpenicillin susceptibility in *S. pneumoniae*. A susceptible oxacillin result indicates penicillin susceptibility, but an oxacillin-resistant result may not reflect true resistance to benzylpenicillin or other beta-lactams and may lead to overestimation of penicillin resistance. In such cases, targeted AST for benzylpenicillin is required to confirm resistance. Abbreviations: BSI: Bloodstream infection; STI: Sexual Transmitted Infection (i.e., urogenital gonorrhoea); UTI: Urinary tract infection; GTI: Gastrointestinal tract infection.

## Data Availability

No new data were created or analyzed in this study. Data sharing is not applicable.

## Abbreviations

The following abbreviations are used in this manuscript:

3GC-R	3rd-generation cephalosporin resistant
AI	Artificial Intelligence
AMR	Antimicrobial Resistance
AMRSNET	Antimicrobial Resistance Surveillance Network
AmpC	AmpC Beta-Lactamase
AST	Antimicrobial Susceptibility Testing
AU	African Union
AWaRe	Access, Watch, Reserve
BSI	Bloodstream infection
CDC	Centre of Disease Control and Prevention
cIAI	Complicated Intra-Abdominal Infection
CMS	Centers for Medicare & Medicaid Services
COVID-19	Coronavirus Disease 2019
CR	Carbapenem-resistant
CRISPR	Clustered Regularly Interspaced Short Palindromic Repeats
cryo-EM	Cryo-Electron Microscopy
cUTI	Complicated Urinary Tract Infection
DALYs	Disability-Adjusted Life Years
DBO	Diazabicyclooctane
EARS-Net	European Antimicrobial Resistance Surveillance Network
EHR	Electronic Health Record
EMA	European Medicines Agency
ESKAPE	Enterococcus faecium, Staphylococcus aureus, Klebsiella pneumoniae, Acinetobacter baumannii, Pseudomonas aeruginosa, and Enterobacter spp.
ESBL	Extended-Spectrum Beta-Lactamase
FDA	Food and Drug Administration
FQ-R	Fluoroquinolone resistant
GAP AMR	Global Action Plan on Antimicrobial Resistance
GLASS	Global Antimicrobial Resistance Surveillance System
HAP/VAP	Hospital-Acquired Pneumonia/Ventilator-Associated Pneumonia
HGT	Horizontal gene transfer
IPC	Infection prevention & control
KPC	Klebsiella pneumoniae Carbapenemase
LMICs	Low- and middle-income countries
MBLs	Metallo-β-lactamases
mcr	Mobilized Colistin Resistance
MDR	Multidrug-resistant
MRSA	Methicillin-Resistant Staphylococcus aureus
NAPs	National Action Plans
NDM	New Delhi Metallo-beta-lactamase
NHSN	National Healthcare Safety Network
PBPs	Penicillin-binding proteins
RiPP	Ribosomally synthesized and Post-translationally modified Peptide
TrACSS	Tracking Antimicrobial Resistance Country Self-Assessment Survey
UHC	Universal health coverage
UTIs	Urinary Tract Infections
WASH	Water, sanitation, hygiene
WGS	Whole-genome sequencing
WHO	World Health Organization
XDR	Extensively drug-resistant
